# Equal Protection? Differential Effects of Religious Attendance on Black-White Older Adult Mortality

**DOI:** 10.1093/geroni/igaa057.1271

**Published:** 2020-12-16

**Authors:** Ellen Idler

**Affiliations:** Emory University, Atlanta, Georgia, United States

## Abstract

Social determinants of later life population health are “the circumstances in which we are born, grow up, live, work, and age” usually identified as power and status determinants: income, wealth, and education. Although rarely considered a social determinant of health, religious social ties are a familiar “circumstance” for many older persons, and there is considerable evidence linking religious attendance to all-cause mortality. There are race differences in both religiosity and mortality patterns: Black Americans show higher levels of both religious attendance and mortality compared with white Americans. This raises the question of equal protection of religious attendance: Is the protective effect of religious attendance on mortality weaker, stronger, or the same for whites and African Americans? The analysis employs 10-year longitudinal data from the Health and Retirement Study, 2004-2014 (N=18,346). In stratified models, after adjustment for sociodemographic factors and health, African Americans have a hazard ratio (HR) for frequent attendance at services that is more protective than for whites: .48 (95%CI: .35, .67) compared with .61 (95%CI: .53, .70). Health behaviors mediate 19% of the effect for blacks and 26% for whites; other social ties mediate 12.5% of the effect for blacks and 7% for whites. An interaction test shows a more protective effect of frequent attendance for blacks compared with whites (p<.000). Religious attendance may be more beneficial for African Americans who are multiply disadvantaged with respect to other social determinants of health. The mediation patterns also suggest that the mechanisms of effect for blacks and whites may differ.

